# Intra-articular Large Ossicle Associated to Osgood-Schlatter Disease

**DOI:** 10.7759/cureus.3008

**Published:** 2018-07-19

**Authors:** Wonchul Choi, Kyunghun Jung

**Affiliations:** 1 Orthopaedics, CHA University/Cha Bundang Medical Center, Sungnam, KOR; 2 Orthopaedic Surgery, CHA University, Seongnam, KOR

**Keywords:** osgood -schlatter disease, ossicle, knee arthroscopy

## Abstract

Osgood–Schlatter disease (OSD) is known as a self-limiting condition but surgical excision of the ossicles may be required in adults resistant to conservative treatments. The ossicle associated to OSD is generally small and located outside the joint near the tibial tubercle; however, large or intra-articular ossicle has been reported rarely. Here, we report an unusual case of OSD with a separated, large-sized ossicle that protruded into the knee joint and treated by arthroscopy-assisted excision of the ossicle.

## Introduction

Osgood–Schlatter disease (OSD) is a common cause of anterior knee pain in adolescents. It is generally a benign and self-limiting disease so conservative treatment usually provides sufficient symptom improvement. Surgical treatment is only indicated if disabling symptoms are unresolved despite adequate conservative treatment. Excision of the ossicle has been regarded as an effective surgical treatment option for refractory OSD cases [1-4]. The ossicle is generally small and locates near the tibial tubercle and beneath the patella tendon. Therefore, activity-related pain focused on the tibial tubercle and distal patellar tendon is a common complaint of OSD patients [5]. However, rarely a large ossicle may protrude into the knee joint [6, 7]. In this case, the site of pain may be different from common cases. Moreover, it is important to distinguish this intra-articular ossicle from tumorous lesions of the infrapatellar fat pad.

Here, we report an unusual case of OSD patient with a separated, large-sized intra-articular ossicle in the infrapatellar fat pad that was treated by arthroscopy-assisted excision of the ossicle.

## Case presentation

A 45-year-old, Caucasian male patient presented with right knee pain that started a year ago and aggravated eight months ago. He was an English teacher and denied a history of any trauma. The symptom sustained despite six months of conservative treatment at another clinic, including intermittent non-steroid anti-inflammatory drug medication, injection (the exact drug is unknown) or physical therapy. He had severe anterior knee pain during stair-climbing or squatting and had been aware of a cystic mass in the posterior aspect of the knee joint that had been progressively increasing in size. On physical examination, mild swelling and effusion of the right knee were seen without localized heat sense. Painful crepitation and moderate tenderness were present around the patellar tendon during knee motion and a large, non-tender cystic mass existed on the posterior aspect of the right knee joint. The crepitation also existed on his left knee, but he had no discomfort on the left side at all. Also, there was no cystic lesion on the left knee. The volume of the right infrapatellar region was greater than normal and felt firm to the touch. Passive full knee range of motion was possible but painful. There was no tenderness on the tibial tubercle or patella.

**Figure 1:**
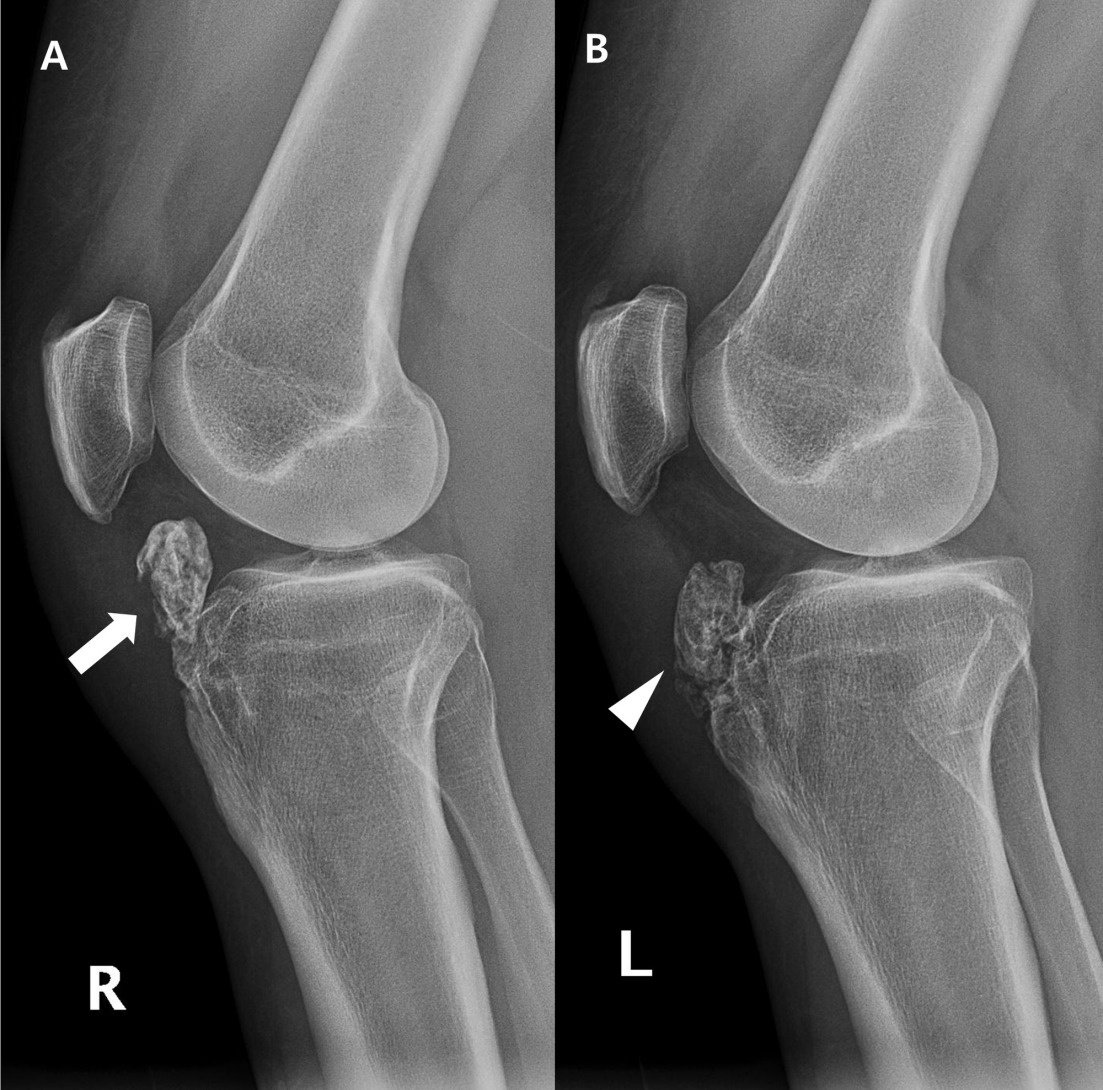
Lateral plain radiographs of both knees. (A) A large osseous lesion (arrow) is seen at the infrapatellar area of the right knee. (B) An osseous body (arrowhead) of equivalent size was partially fused to the hypertrophied tibial tuberosity of the left knee.

Plain radiographic examination of the right knee showed a large ossicle locating beneath the patellar tendon and inside the infrapatellar fat pad. A similar-sized ossicle was seen in the left knee, while it was partially fused to the hypertrophied tibial tuberosity (Figure 1).

**Figure 2:**
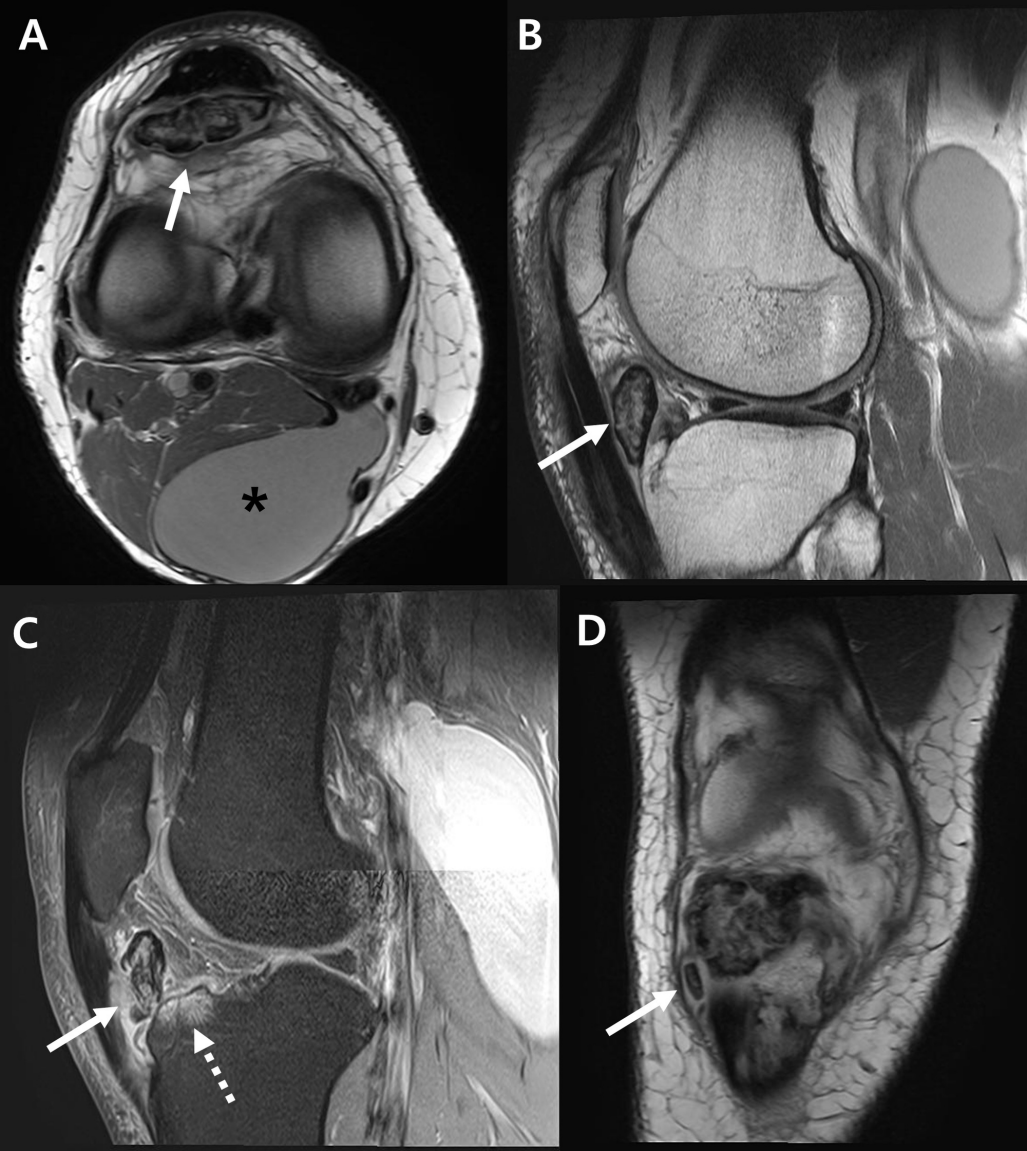
Right knee magnetic resonance images. (A) A large ossicle with heterogenous signal intensity (arrow) and a Baker’s cyst (asterisk) are seen on axial image. (B) From T1-weighted sagittal image, the ossicle (arrow) is separately located inside the infrapatellar fat pad. (C) T2-weighted sagittal image shows inflammation of patellar tendon (solid arrow) and bone marrow edematous change of anterior tibia plateau (dotted arrow). (D) Small separated ossicle (arrow) was found inferior to the large ossicle from coronal image.

Right knee magnetic resonance images showed about 3.8 x 1.3 x 3.0 cm sized, well-circumscribed ossicle. Several smaller ossicles were also found near the tibial tubercle. Inflammatory changes in synovium, infrapatellar fat pad, and patellar tendon were also seen. A reactive bone marrow edema was found at the anterior aspect of the tibial condyle. Besides, a Baker’s cyst of 7.3 x 4.1 x 14.7 cm size was found (Figure 2).

**Figure 3:**
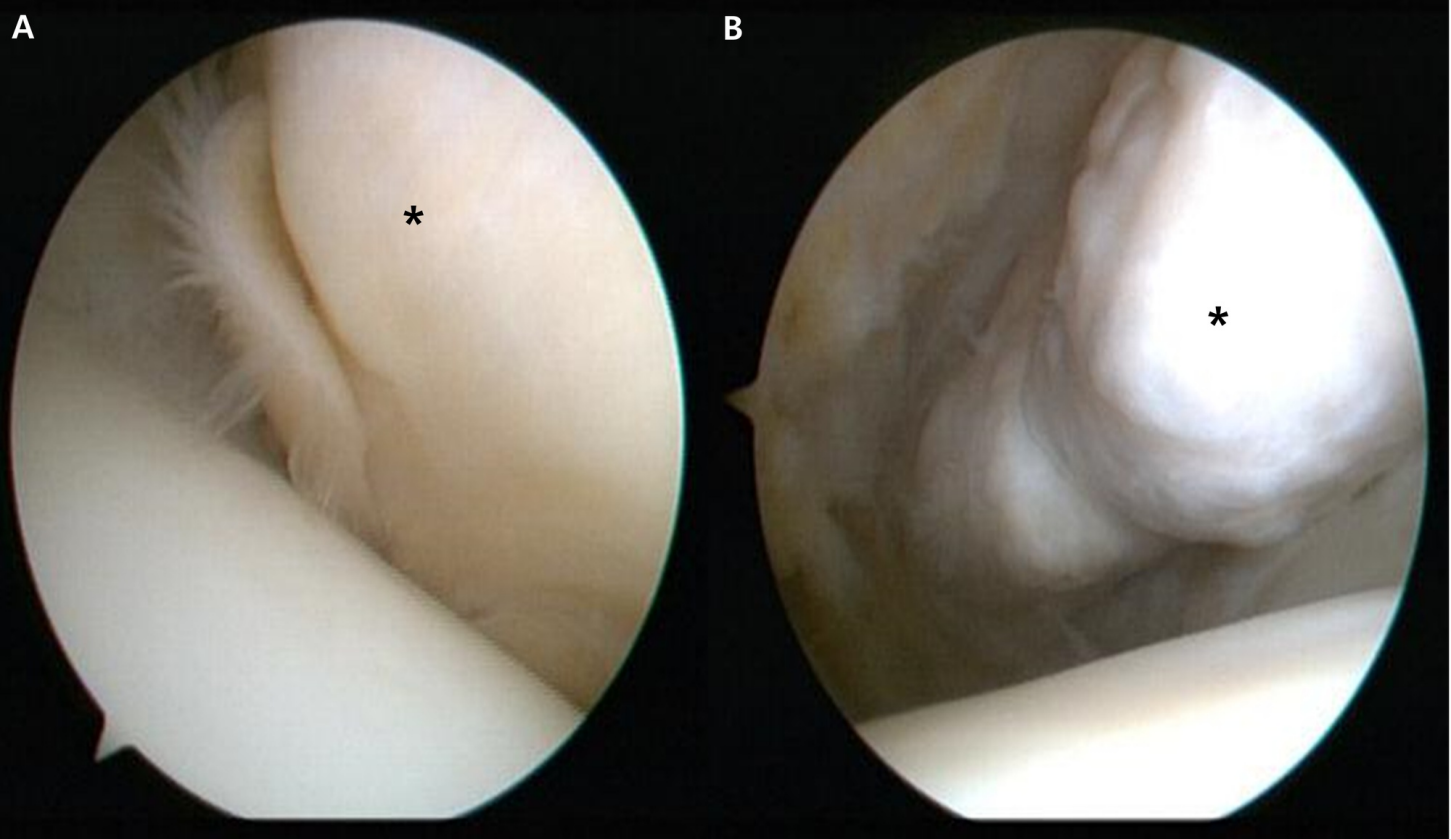
Right knee arthroscopy findings. (A) The ossicle was covered by soft tissue (asterisk). (B) After removing the soft tissue by mechanical shaver, the ossicle (asterisk) is exposed.

The patient chose to get surgery since the symptom continued after the prolonged conservative treatment. Since the lesion was benign in character and the patient requested to remove the Baker’s cyst simultaneously, arthroscopic surgery was planned to excise the ossicle and decompress the cyst. Right knee arthroscopy was performed through the standard anterolateral and anteromedial portals close to the patellar tendon. Anterior interval release of the hypertrophied fat pad was carefully performed with the mechanical shaver. Then the arthroscopy was inserted through a separate superolateral portal. The knee was held in a 45-degree flexed position and the outline of the ossicle was identified. The overlying fat and fibrous tissues were shaved, and the clear outline of the ossicle was visible (Figure 3).

**Figure 4:**
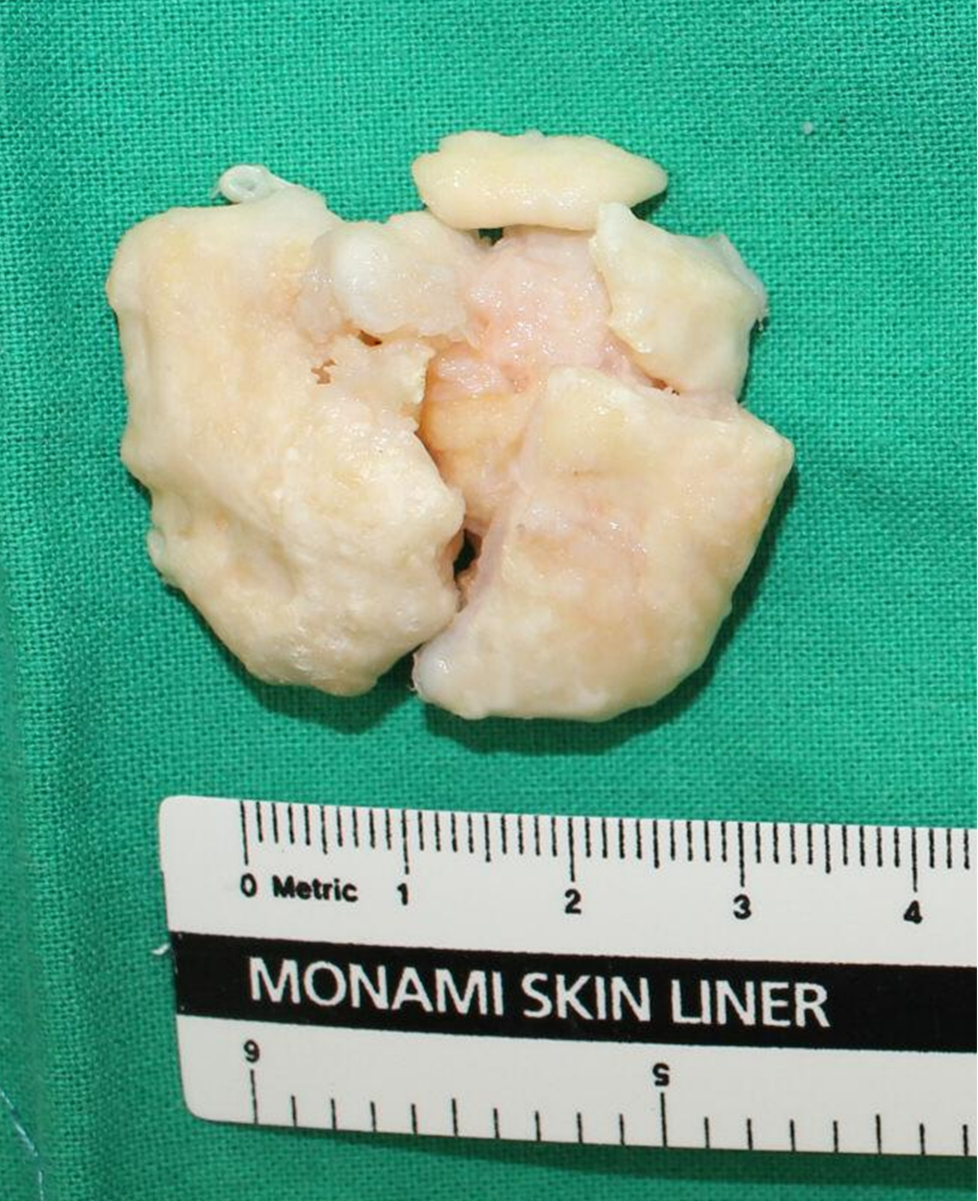
Gross appearance of the excised ossicle. The ossicle was fragmented during surgery.

The ossicle was fragmented using an arthroscopic punch or a small osteotome and removed through the slightly extended anteromedial portal (Figure 4).

**Figure 5:**
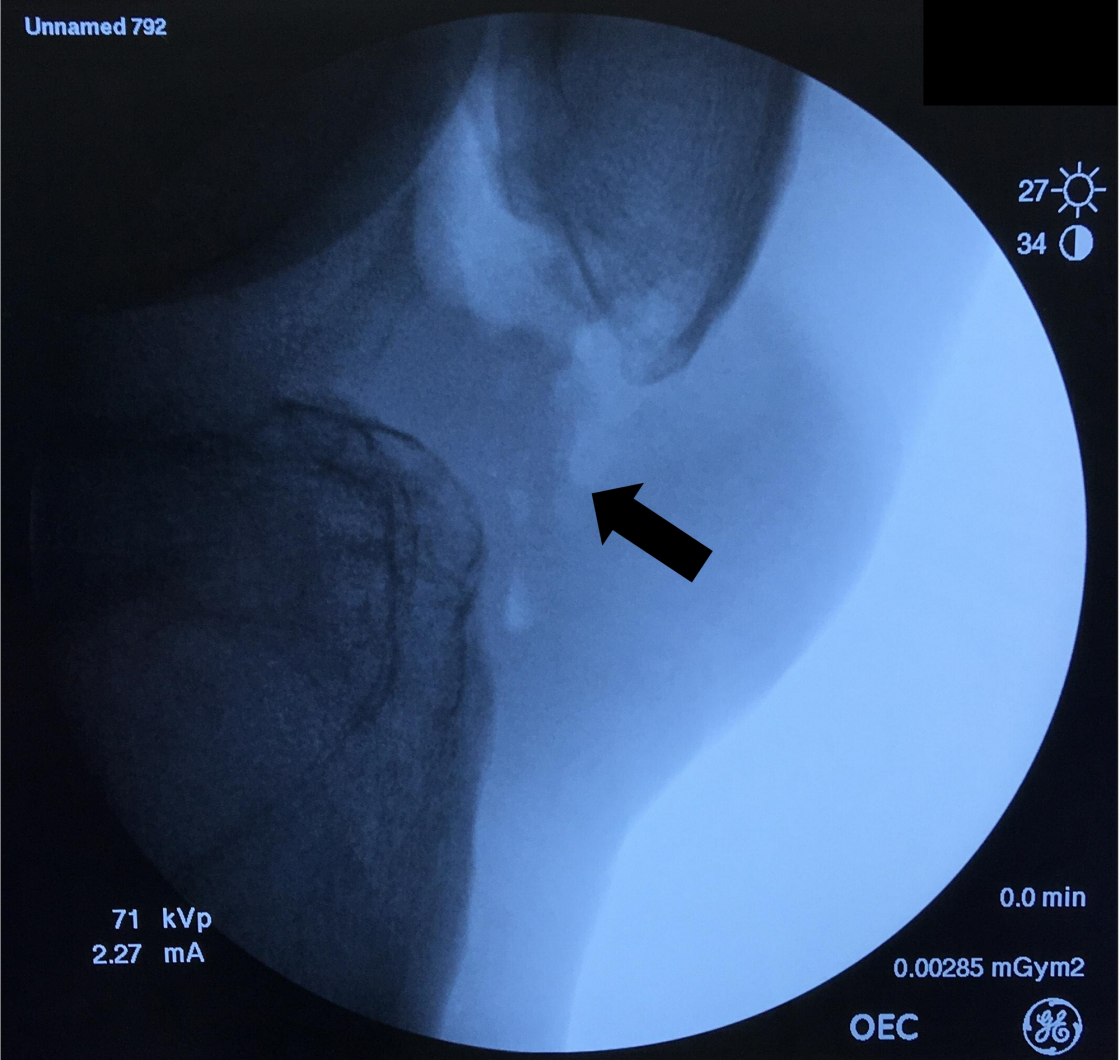
Intraoperative fluoroscopy finding. Complete removal of the ossicle was confirmed (arrow).

An intraoperative fluoroscopic image was acquired to confirm the complete removal of the ossicle (Figure 5).

The knee was held in a 15-degree flexed position and the inflamed retro-patellar tendon surface was debrided. Through the additional posteromedial portal, the Baker’s cyst was decompressed.

**Figure 6:**
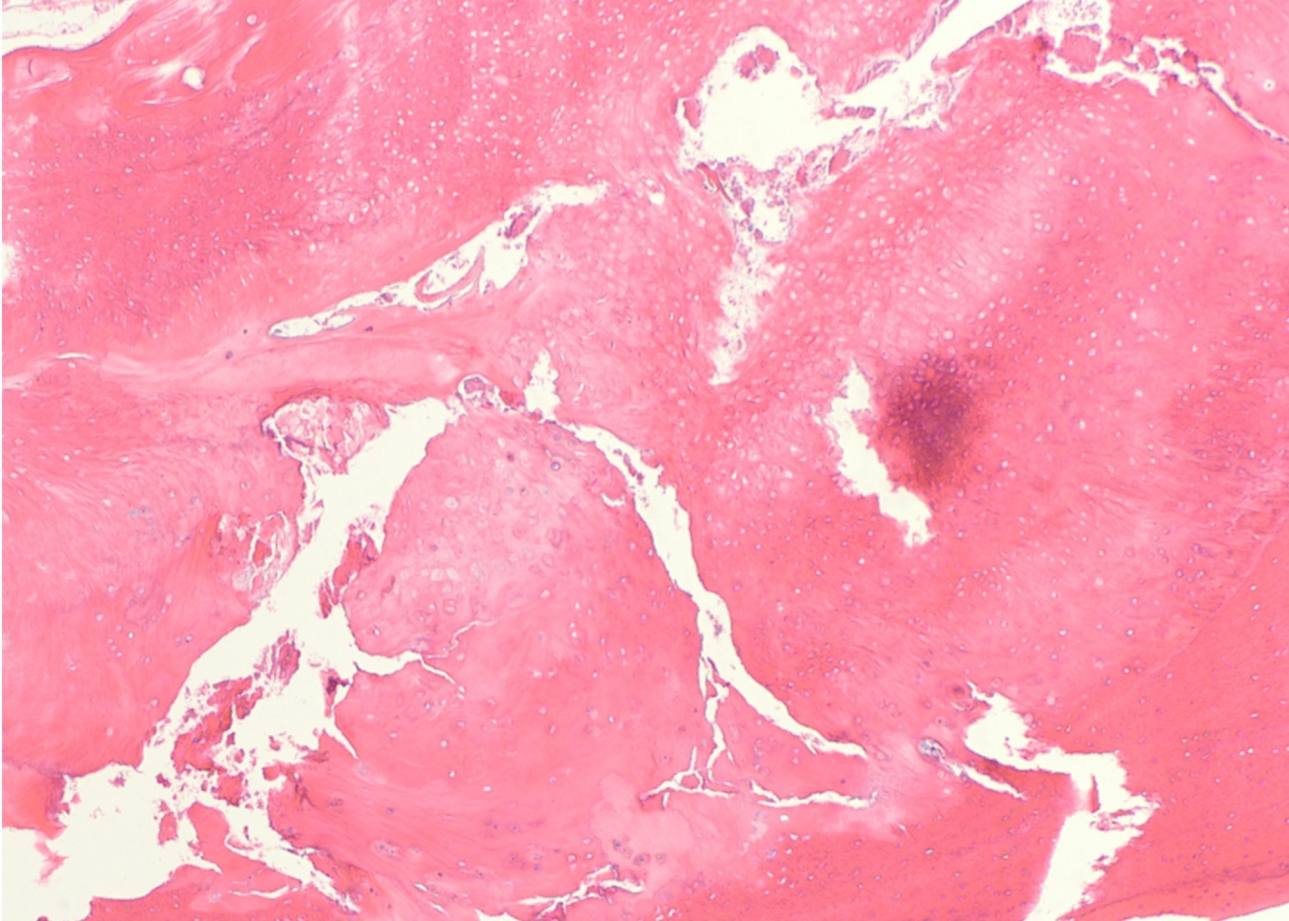
Histopathology finding. The hematoxylin and eosin staining showed osteocartilaginous tissue without distinct cartilage cap, suggestive of ossicle associated to Osgood-Schlatter disease.

Postoperative recovery was uneventful. From the first postoperative day, the patient could walk with full weight-bearing and move his knee. Strengthening exercise for knee extensor muscles was encouraged as tolerable. The patient regained full knee range of motion and returned to his daily activities within two weeks after surgery. The excised tissue was subjected to a histopathological examination. The histology finding was consistent with OSD that mix of osseous and cartilaginous tissue was seen without evident cartilaginous cap (Figure 6). At six months after the surgery, the patient’s right knee was normal without any clinical or radiographic sign of recurrence.

## Discussion

OSD is known as a traction apophysitis of the tibial tuberosity which caused by the stress from the patellar tendon at its insertion [8, 9]. OSD is one of the most common causes of anterior knee pain in adolescents, but the symptom generally ceases with skeletal maturity [10]. In some cases, the symptom remains until adult life but usually alleviates by non-operative treatment. So, surgical treatment is only indicated when the symptom persists even after adequate conservative treatment.

The patient of our case report had sustained pain despite six months of conservative treatment, so it was common and reasonable to consider surgery. However, the clinical features were unusual that the size of ossicle was larger than common OSD and the location was inside the joint. Also, the symptom was different from common OSD that painful crepitation was present during knee motion and the tender point was on the infrapatellar fat pad area instead of the tibial tuberosity area. It is notable that similar lesion of the left knee did not cause any symptom. There was a difference that the ossicle of the right knee was separated, while it was partially fused to the tibia on the left side. It is possible that mobile osseous fragment caused irritation on the surrounding tissue and resulted in the soft tissue inflammation and the reactive bone change [11]. The patient had a large Baker’s cyst along with the ossicle in the right knee. It is uncertain if those two lesions are related to each other. However, it is possible that the inflammation caused by the intra-articular ossicle has increased the joint fluid and resulted in the enlargement of Baker’s cyst.

Although it is uncommon to find such a large, intra-articular ossicle, the most likely diagnosis was OSD considering that the similar lesions have involved bilateral knees. Differential diagnosis included benign tumorous lesion of the infrapatellar fat pad such as synovial chondromatosis or osteochondroma [12-14]. Calcification combined with Hoffa’s disease was also considered [15]. With low probability, the possibility of malignant lesion including chondrosarcoma or synovial sarcoma had to be ruled out.

There were successful reports about open [4, 11, 16] or arthroscopic [2, 3, 6] excision of ossicle and debridement of tibial tuberosity for the treatment of OSD, but care must be taken not to violate the patellar tendon. Recently, Pagenstert et al. have reported a satisfactory result after treatment of refractory OSD by reduction osteotomy of tibial tubercle [17]. Also, there was a case report showing the effect of repetitive percutaneous screw fixation of tibial tuberosity for adolescent OSD [18]. However, it should be noted that most of the studies were case reports with small subject numbers. There is no standardized surgical treatment yet, so the surgeon needs to choose one that is appropriate for each situation. Since the ossicle was located inside the knee joint and had a benign feature, arthroscopic excision was effective in our case. Arthroscopic view from superolateral portal may be helpful to visualize and remove the ossicle if the lesion is large and located inside the infrapatellar fat pad.

## Conclusions

Although it is rare, intra-articular presentation of large ossicle associated to OSD is possible. In this case, differential diagnosis including benign tumorous condition or calcification of infrapatellar fat pad should be considered. The excision of ossicle can be an effective treatment option if the symptom persists despite adequate non-operative treatment. In case of intra-articular ossicle presentation, arthroscopy-assisted removal may be effective.
